# Developmental expression of inhibitory synaptic long-term potentiation in the lateral superior olive

**DOI:** 10.3389/fncir.2014.00067

**Published:** 2014-06-19

**Authors:** Vibhakar C. Kotak, Dan H. Sanes

**Affiliations:** ^1^Center for Neural Science, New York UniversityNew York, NY, USA; ^2^Department of Biology, New York UniversityNew York, NY, USA

**Keywords:** GABA, glycine, GABA_B_, synaptic potentiation, plasticity, LSO

## Abstract

Principal neurons of the lateral superior olivary nucleus (LSO) respond selectively to interaural level differences (ILD). To perform this computation, LSO neurons integrate excitatory synaptic drive from the ipsilateral ear with inhibitory synaptic drive from the contralateral ear via the medial nucleus of the trapezoid body (MNTB). Previous research demonstrated that inhibitory terminals from the MNTB to the LSO are eliminated during development. Furthermore, MNTB synapses display an activity- and age-dependent long-term depression (iLTD) that may contribute to inhibitory synapse elimination. However, inhibitory synapses that are stabilized become stronger. Here, we asked whether MNTB synapses displayed activity-dependent strengthening. Whole-cell recordings were obtained from LSO neurons in a gerbil brain slice before and after hearing onset. The inhibitory MNTB afferents were stimulated at a low rate, similar to spontaneous discharge rates observed *in vivo*. The MNTB-evoked inhibitory responses were strengthened by 40–300% when synaptic activity was coupled with postsynaptic membrane depolarization, exogenous glutamate application, or activation of ipsilateral excitatory synaptic inputs. This inhibitory long-term potentiation (iLTP) was associated with increased spontaneous inhibitory postsynaptic current (IPSC) amplitude and frequency. One hour after iLTP induction, IPSCs could not be de-potentiated by the MNTB stimulation pattern that induces iLTD in control slices. iLTP could only be induced after hearing onset (>P12), and was blocked in the presence of a GABA_B_ receptor antagonist. Together, these results suggest a developmental period during which the induction of iLTP depends on the conjoint activation of GABA_B_ receptors and postsynaptic depolarization. We propose that iLTP may support stabilization of un-pruned MNTB connections and contribute to the emergence of ILD processing in the mature LSO.

## Introduction

The encoding of sound localization cues, such as interaural level (ILD) and time differences (ITD), begins in the ventral auditory brain stem. For ILD coding, the discharge rate of lateral superior olivary (LSO) neurons is proportional to the integration of ipsilateral excitatory drive arising from the cochlear nucleus and contralateral inhibitory drive from the medial nucleus of the trapezoid body (MNTB) (Boudreau and Tsutchitani, 1968; Caird and Klinke, 1983; Harnischfeger et al., 1985; Tollin, 2003; Sterenborg et al., 2010). The inhibitory MNTB afferents form a tonotopic projection in the LSO that is aligned precisely with the ipsilateral excitatory projection in the adult (Sanes and Rubel, 1988). Furthermore, this precision evolves as inhibitory synapses are eliminated during postnatal development (Sanes and Siverls, 1991; Sanes and Friauf, 2000; Kim and Kandler, 2003; Kandler, 2004; Kandler and Gillespie, 2005; Kandler et al., 2009). A similar elimination of inhibitory MNTB terminals occurs at the medial superior olivary nucleus, which encodes ITD (Kapfer et al., 2002), although one study did not find a significant developmental change in amplitude (Walcher et al., 2011). It is, therefore, plausible that that the establishment of properly aligned excitatory and inhibitory maps involves the dynamic addition and elimination of inhibitory synapses. One mechanism that could participate in synapse elimination, inhibitory long-term depression (iLTD), has been described previously for MNTB synapses (Kotak and Sanes, 2000, 2002; Kotak et al., 2001; Chang et al., 2003). As inhibitory synapses are eliminated and the remaining contacts are strengthened in the rat LSO, there is a 12-fold increase in inhibitory conductance (Kim and Kandler, 2003). Here, we describe a mechanism that could account for this strengthening.

A vast literature on excitatory long-term potentiation (LTP) and depression (LTD) in the hippocampus and neocortex supports their involvement in adult learning, memory and neurodevelopmental disorders (see Bear and Malenka, 1994; Bliss et al., 2013). Many studies have also shown that inhibitory synapses can be strengthened or weakened in an activity-dependent manner. However, the functional consequence of such inhibitory synapse plasticity and the underlying biochemical and molecular factors are not well understood. Inhibitory plasticity, including GABAergic LTP and LTD, may contribute to memory formation or motor learning (Morishita and Sastry, 1996; Aizenman et al., 1998; Ouardouz and Sastry, 2000). Glycinergic LTP at the goldfish Mauthner neuron may dampen the escape response (Oda et al., 1995, 1998), and GABAergic LTP in the visual cortex may alter visual coding properties (Komatsu and Iwakiri, 1993; Komatsu, 1994, 1996; Komatsu and Yoshimura, 2000). Since MNTB-mediated iLTD gradually wanes following hearing onset (Kotak and Sanes, 2000), it is possible that iLTP emerges during this time. Our results demonstrated that even very low levels of inhibitory afferent activity, when coupled with excitatory transmission, can trigger iLTP after hearing onset. Thus, early binaural cues may be critical in consolidating the functional maturation of LSO inhibitory synapses.

## Materials and methods

**Figure 1:**
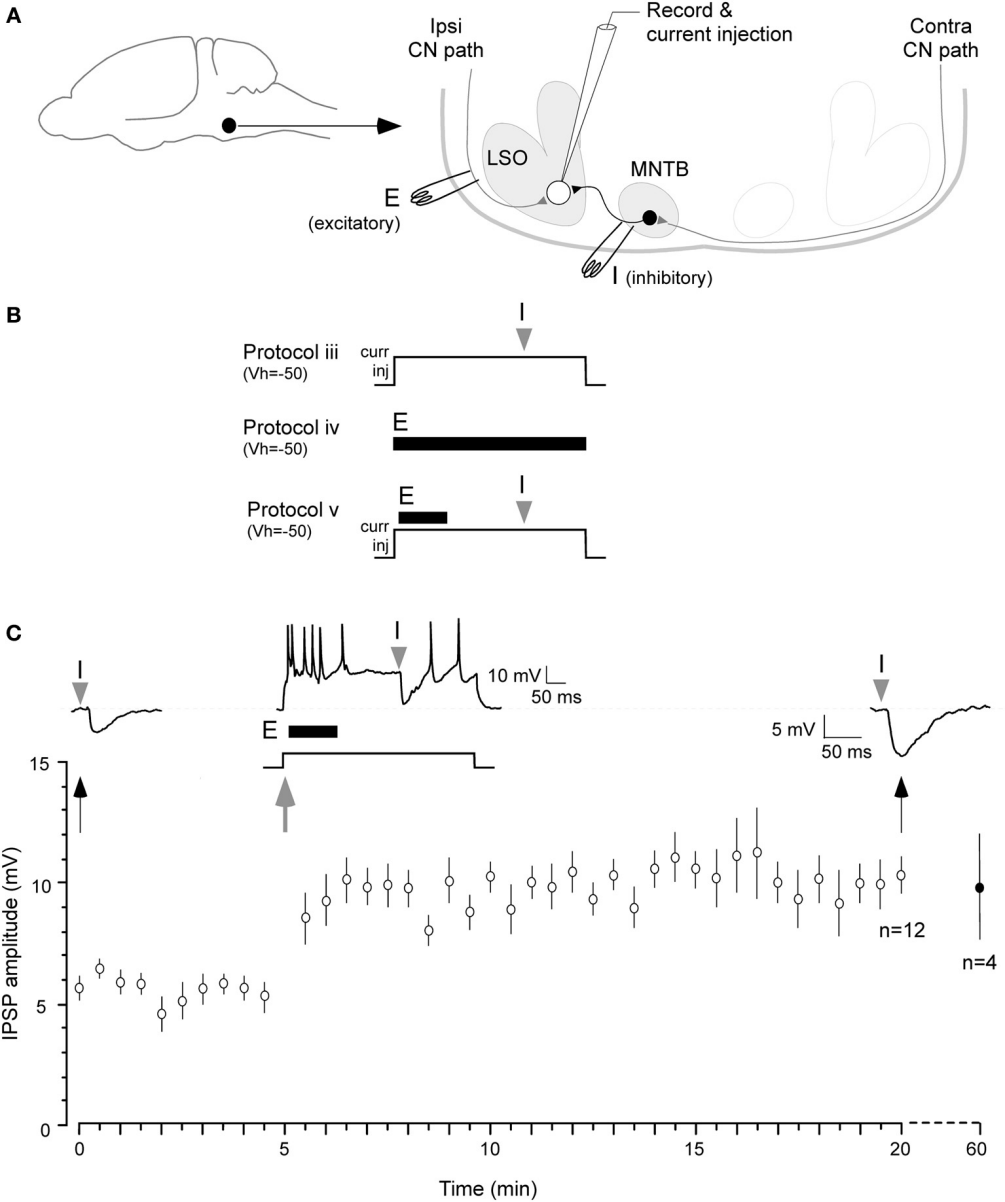
**Iaqnduction of inhibitory LTP in P12–15 day neurons**. **(A)** A schematic of the LSO circuit in brain slice preparation. Filled circle and arrow indicate auditory brainstem. E: ipsilateral excitatory pathway from cochlear nucleus (CN), I: MTNB pathway activated by contralateral CN. **(B)** Three of the stimulus protocols are shown (see Results for details). Only Protocol v was effective in inducing iLTP, as shown in **(C)**. **(C)** A pre-conditioning MNTB-evoked IPSP evoked by MNTB stimulus (I). Such IPSPs were obtained at 0.033 Hz for 5 min at *V*_HOLD_ = −50 mV (baseline). A single suprathreshold depolarization (500 ms, +5–10 pA) injection was used to trigger neuronal firing by up to 50 Hz. The ipsilateral excitatory afferents were then stimulated at 100 Hz 10 ms after the onset of 500 ms depolarization (10 pulses, dark bar under the trace, E). MNTB evoked IPSP was timed at 300 ms after the injection of the 500 ms depolarizing pulse (gray arrowhead). The 0.033 Hz acquisition of IPSP was continued through the recording session. IPSPs displayed a significant enhancement in amplitude for at least 20 min (top right trace 20 min after). Bottom panel shows summary data (mean ± s.e.m.). In 4 neurons, recordings were continued for an additional 40 min, and the increased amplitude IPSPs persisted. IPSP means (Y axis) are sub-maximum amplitude and not normalized.

All protocols were reviewed and approved by the New York University Institutional Animal Care and Use Committee. Gerbil pups *(Meriones unguiculatus)* aged postnatal (P) days 7–15, were used to generate 300 μm transverse brain slices containing the MNTB-LSO circuit (Sanes, 1993). The artificial cerebrospinal fluid (ACSF) contained (in mM): 125 NaCl, 4 KCl, 1.2 KH_2_PO_4_, 1.3 MgSO_4_, 24 NaHCO_3_, 15 glucose, 2.4 CaCl_2_, and 0.4 L-ascorbic acid (pH = 7.3 when bubbled with 95% O_2_/5% CO_2_). ACSF was continuously superfused in the recording chamber at 3 ml per min at 32 ± 1°C. Whole-cell current clamp or voltage or recordings were obtained from LSO neurons (Warner PC-501A) and 200 μs current pulses were delivered directly to the MNTB via bipolar stimulating electrodes to elicit IPSPs or IPSCs, respectively (Kotak et al., 1998). Ipsilateral excitatory afferents were stimulated by a separate bipolar stimulating electrodes at specific frequencies (Results) (Figure 1). Recording electrodes were fabricated from borosylicate glass microcapillaries (1.5 mm OD), and when filled with internal solution the resistance was 5–15 MΩ. For current clamp recordings, the internal patch solution contained (in mM): 127.5 mM potassium gluconate, 0.6 EGTA, 10 HEPES, 2 MgCl_2_ 5 KCl, 2 ATP, and 0.3 GTP. For both internal solutions, the pH of was adjusted to 7.2 with KOH. For voltage clamp recordings, the internal solution was similar to current clamp solution (follows) except potassium gluconate was replaced by an equimolar concentration of cesium gluconate to block voltage-dependent potassium channels, and QX-314 (5 mM) was added to block voltage-dependent sodium channels. The pH was adjusted to 7.2 with CsOH. Further, kynurenic acid (4 mM) was added to the ACSF to block ionotropic glutamate receptors (Moore et al., 1998). Access resistance was balanced throughout the recordings and ranged between 10 and 40 MΩ.

All data were collected using a Macintosh G4 platform running a Mac OS X compatible custom IGOR (WaveMetrics, v3.5) macro called SLICE. The data were analyzed off-line using a second IGOR macro called SLICE ANALYSIS (Kotak et al., 2001). Custom algorithms were used to measure the amplitude and frequency of sIPSCs (Kotak et al., 2005). Data values are presented as mean and standard error of the mean (SEM). Initial IPSP/IPSC amplitudes were compared vs. IPSC amplitudes at the end of the recording session with a non-parametric test (Wilcoxon; Kotak and Sanes, 2000). All statistical analyses were performed using the SAS-based statistical software (JMP v5.0).

## Results

The data in this paper were collected from 60 principal neurons from 42 animals located in the high frequency medial limb of the LSO, each from a separate brain slice. We first asked whether iLTP could be induced under normal physiological conditions in a brain slice preparation without any intracellular or pharmacological manipulations. These current clamp recordings were similar to those employed for the induction of iLTD as described previously (Kotak and Sanes, 2000) except the stimulation rates and postsynaptic depolarization was different.

### Current clamp recordings

First, to determine whether IPSPs recorded in current clamp without glutamate receptor antagonists exhibited any change in strength, we attempted the following protocols. The stimulus to evoked IPSPs was first calibrated to evoke an IPSP at 50% of its maximum amplitude. This enabled us to observe the possible expression of either potentiation or depression. To do this, MNTB was stimulated in incremental intensity (200 μs, 5 μA increments) until maximum amplitude IPSP was obtained. We then selected the stimulus intensity that evoked an approximately 50% maximum amplitude (200 μs, ~80–90 μA).

*Protocol i*: IPSPs were recorded at a rate of 0.033 Hz for approximately 1 h. Under this condition, no change in amplitude was observed (initial IPSP amplitude: 7.4 ± 0.8 mV vs. IPSP amplitude at 60 min: 6.3 ± 0.7 mV; *t* = 2.1, *p* > 0.05; *n* = 7). *Protocol ii*: Our next step was to test MNTB stimulation rates of 0.1 or 5 Hz. These rates were dissimilar to the rate that produced iLTD (1 Hz, 15 min) in our previous study. Under these conditions, no significant change in IPSP amplitude was observed for up to 10 min (not shown). *Protocol iii*: LSO neurons were held slightly more depolarized (at −50 mV) than their resting membrane potential (ranged between −51 and −55 mV) by injection of a small DC current (+5–10 pA). This provided a steady membrane potential baseline against which inhibitory strength could be recorded. In addition, a suprathreshold depolarization (500 ms) was used to increase neuronal firing by up to 50 Hz, a firing rate that may elevate intracellular calcium to support induction of iLTP. Furthermore, a single MNTB-evoked IPSP was timed to occur 300 ms after the onset of postsynaptic depolarization. This regimen was ineffective in triggering iLTP (*n* = 4, not shown). *Protocol iv*: To test whether glutamatergic transmission was sufficient to induce iLTP, ipsilateral excitatory afferents were stimulated at 20 Hz (10 pulses) while cells were held at −50 mV. This protocol did not induce a significant change in IPSP amplitude (*n* = 4, not shown). Since each of these protocols was ineffective, we predicted that the induction of iLTP would require a greater level of postsynaptic glutamate receptor activation.

The protocol that proved to be effective, involved a combination of the above manipulations. *Protocol v*: Cells were held at −50 mV and a single 500 ms postsynaptic depolarization to induce up to 50 Hz firing was elicited with current injection. An increased level of ipsilateral excitatory afferent stimulation (100 Hz, 10 pulses) was timed to occur 10 ms after the onset of this depolarization. Lastly, MNTB stimulation was timed to occur 300 ms after the onset of the 500 ms postsynaptic depolarization (Figure 1, top panel). Under these conditions, IPSPs were potentiated for 20 min or longer (Figure 1C; initial IPSP amplitude: 5.9 ± 0.3 mV, IPSP amplitude 20 min after conditioning: 10.1 ± 0.6 mV; Wilcoxon test, *X*^2^ = 3.9, *p* = 0.001, *n* = 7). In 4 of these cases, when the recording session was extended, increased IPSP amplitude persisted for 1 h after the conditioning protocol (9.7 ± 1.95 mV).

**Figure 2:**
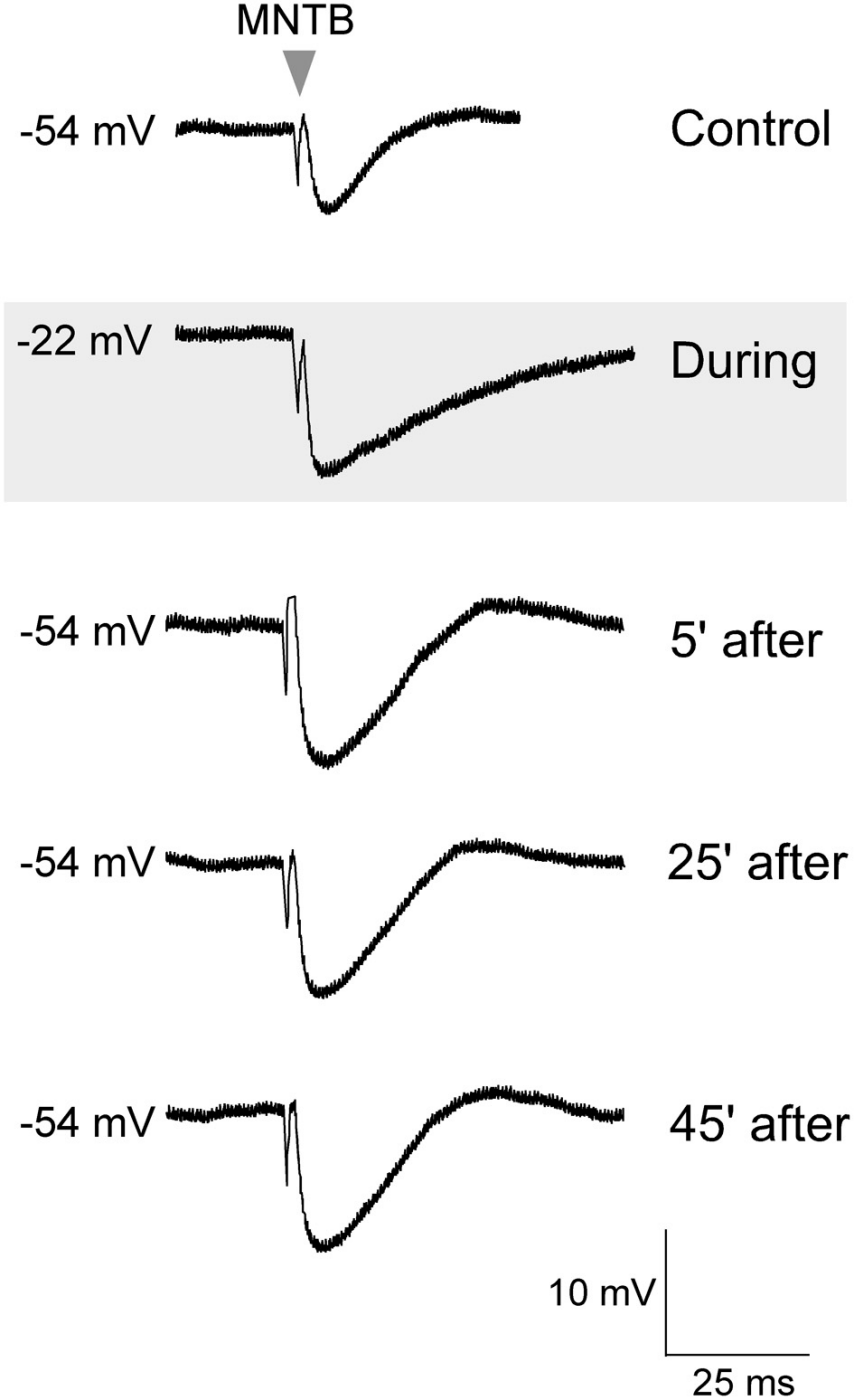
**Glutamate exposure potentiated MNTB-evoked IPSPs**. Control MNTB-evoked IPSPs were recorded at 0.033 Hz. An initial response is shown in the top trace. While continuing with this stimulation, glutamate (10 mM) was bath perfused (45 s), and the neuron became depolarized (gray panel), and discharged intensely (not shown). After the cell had completely repolarized to its original resting potential, the IPSP amplitudes were significantly larger. Baseline IPSPs (pre glutamate) and those acquired during glutamate exposure and then at 5, 25, and 45 min after the cell had repolarized are shown.

To test whether such co-activation of ipsilateral excitatory afferents could have involved postsynaptic activation of glutamate receptors to induce iLTP, in separate experiments, glutamate was bath applied (10 mM, 45 s) in the absence of ipsilateral afferent stimulation (Kotak and Sanes, 1995). Thus, this protocol was designed to bypass the possible co-recruitment of ipsilaterally evoked inhibition (Kotak and Sanes, 1997). Sub-maximum IPSPs were first elicited for 10 min to establish a baseline, before the application of glutamate. Glutamate treatment depolarized the LSO neurons from none to by up to 20 mV, and increased the discharge rate of recorded neurons from none up to 50 Hz. The MNTB stimulation was maintained during this depolarization for 30 min and MNTB stimulation continued after the cells had returned to the starting *V*_REST_. As shown in Figure 2, when IPSPs were recorded after complete recovery of the membrane potential, we observed significantly enhanced IPSP amplitudes (IPSP pre-glutamate treatment: 8.4 ± 0.12 mV vs. IPSP 30 min after the recovery of membrane potential, 12.6 ± 0.2 mV; Wilcoxon test, *X*^2^= 3.8, *p* < 0.05, *n* = 3).

### Voltage clamp recordings

**Figure 3:**
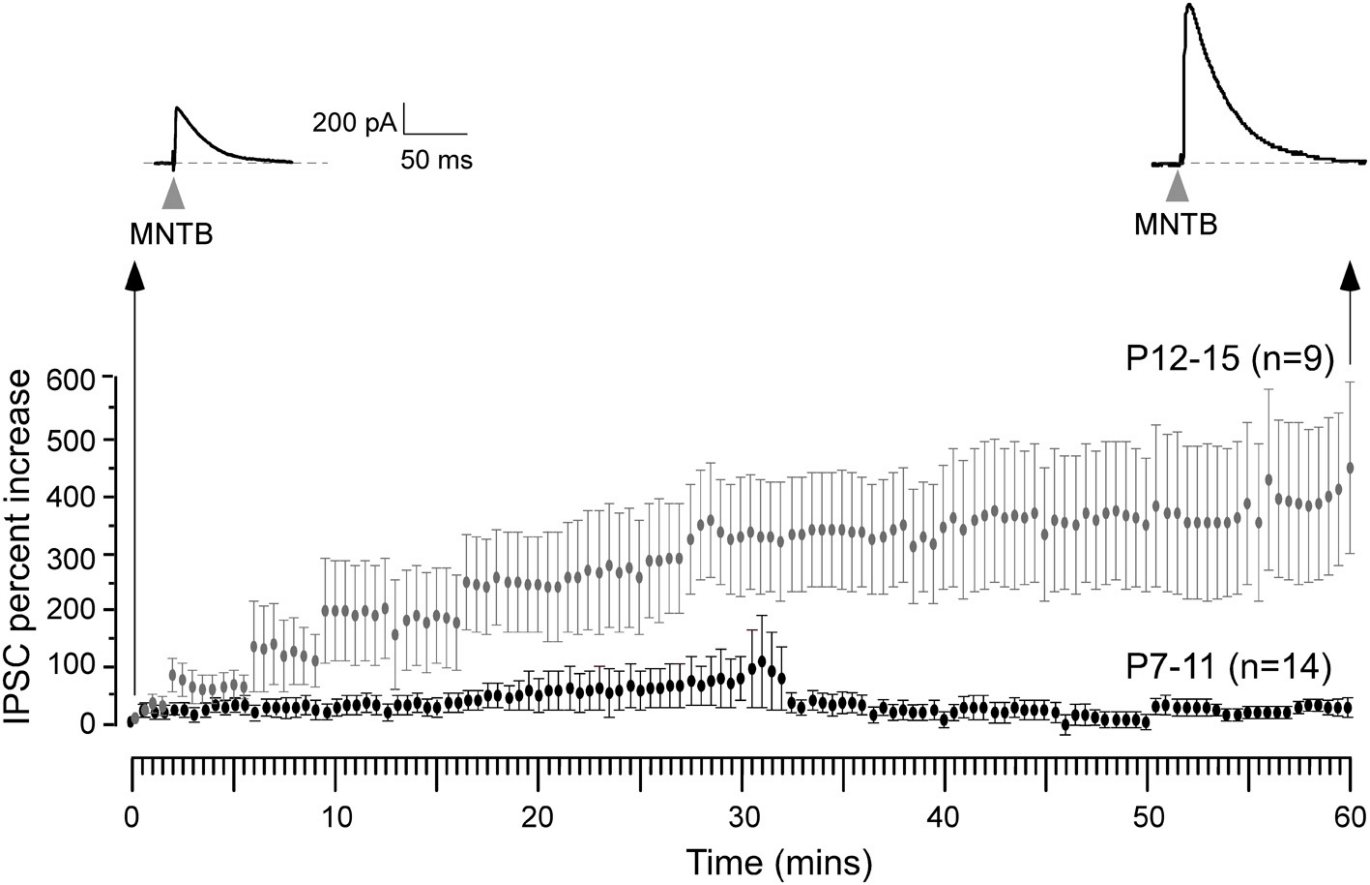
**Expression of iLTP is age-dependent**. MNTB was continuously stimulated at 0.033 Hz for 1 h while voltage clamp recordings were obtained from the LSO neurons in the presence of ionotropic glutamate receptor antagonist kynuranic acid at *V*_HOLD_ = 0 mV. Under these circumstances, IPSCs were recorded as outward currents. An IPSC recorded 1 h after this stimulation protocol shows a dramatic enhancement in its amplitude (top right trace) when compared to the initial IPSC (top left trace). Long-term potentiation of IPSCs was observed for neurons recorded at P12–15 (gray symbols), but not at P7–11 (black symbols).

We then asked whether iLTP could be induced by stimulation of the MNTB afferents under voltage clamp conditions (*V*_HOLD_ = 0 mV, ionotropic glutamate receptors blocked, thus in an absence of ipsilateral afferent stimulation) identical to the recording conditions employed for experiments in which iLTD was induced (Kotak and Sanes, 2000). For iLTD induction, we had used low frequency stimulation paradigm (LFS, 1 Hz for 15 min) that led to long-term inhibitory depression for at least an hour. We could also induce and perturb iLTD in current clamp conditions using similar LFS regimen (Kotak et al., 2001). In pilot voltage clamp recordings when the MNTB was stimulated at a very low rate (0.033 Hz) to monitor baseline inhibitory strength we observed a small increase in IPSC amplitude. Therefore, we chose to carry out a full set of experiments using 0.033 Hz. Continuous stimulation of the MNTB at 0.033 Hz produced a gradual increase in IPSC amplitude, and this enhancement became progressively larger during the recording session. As shown in Figure 3, neurons from P12–15 animals displayed a ~400% increase in IPSC amplitude over the course of 1 hr, as compared to the initial baseline values (initial IPSC: 156 ± 26 pA vs. IPSC at 10 min of stimulation: 224 ± 74 pA, Wilcoxon test, *X*^2^ = 5, *p* = 0.02; initial IPSC: 156 ± 26 pA vs. IPSC at 30 min of stimulation: 472 ± 90, *X*^2^= 7.7, *p* = 0.005; initial IPSC: 156 ± 26 pA vs. IPSC at 60 min of stimulation: 495 ± 94, *X*^2^= 8.7, *p* = 0.003; *n* = 9). When an identical stimulation protocol was employed on neurons from P7 to 11 animals, no significant change in IPSC amplitude was observed for up to 60 min (Figure 3). (Initial IPSC: 220 ± 68 pA vs. IPSC at 10 min of stimulation: 194 ± 54 pA, IPSC at 30 min of stimulation: 227 ± 58 pA, IPSC at 60 min of stimulation: 198 ± 67 pA, Wilcoxon test, *p* > 0.05 for each comparison; *n* = 14).

For controls, we have previously shown that the baseline of evoked IPSPs or IPSCs were stable through the entire 90 min recording session (see Figure 2 in Kotak and Sanes, 2000; Kotak et al., 2001). In the current study, whereas stimulation at 0.033 Hz produced robust iLTP after hearing onset, identical recording conditions and stimulation rates did not lead to any change in baseline sIPSCs before hearing onset (see Figure 3). Therefore, we did not perform additional controls. Voltage clamp recordings, similar to conditions in our previous iLTD studies (Kotak and Sanes, 2000; Chang et al., 2003), cells were held depolarized at 0 mV and 4 mM kynurenic acid was added to the ACSF (pH 7.3 after bubbling with O_2_/CO_2_) to block ionotropic AMPA and NMDA receptors. Spontaneous IPSCs (sIPSC) too were recorded as outward currents before and after the stimulation protocol at *V*_HOLD_ = 0 mV. The very low frequency stimulation paradigm (0.033 Hz) was then applied continuously through the recording session (1 h) both to induce plasticity as well as acquire IPSCs.

**Figure 4:**
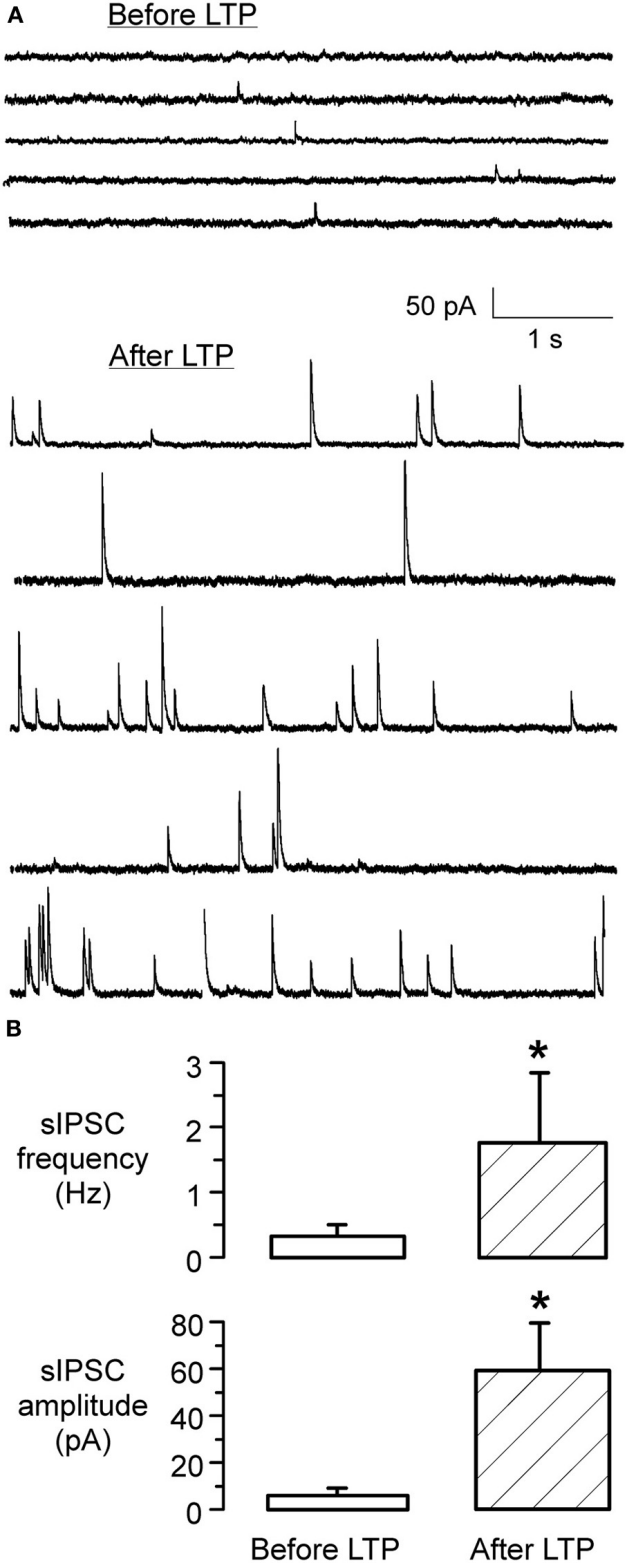
**iLTP is associated with increased sIPSC frequency and amplitude. (A)** sIPSCs recorded in an LSO neuron before the induction of iLTP (top) displayed a low frequency and small amplitudes. In contrast, sIPSCs recorded from the same neuron after the induction of iLTP, during the last 5 min of the recording session, displayed a dramatic increase in frequency and amplitude (bottom). **(B)** Bar graph shows the significant increase in sIPSC frequency 1 h following the induction of LTP (*p* < 0.001) (top). Similarly, there was a dramatic increase in the mean amplitude of sIPSCs (*p* = 0.01) (bottom).

To assess whether there was any alteration in the presynaptic release properties following the conditioning protocol, spontaneous IPSC (sIPSC) amplitude and frequency were monitored at early (initial 10 min) and late (60 min) periods of the iLTP expression. As shown in Figure 4, these data indicate that iLTP was accompanied by a large increase in both the frequency and amplitude of spontaneous IPSCs (sIPSC amplitude before LTP: 6.3 ± 1.8 pA vs. sIPSC amplitude 60 min afterLTP: 61 ± 19.5 pA; Wilcoxon test, *X*^2^= 7.8, *p* < 0.001; sIPSC frequency before LTP: 0.28 ± 0.14 Hz vs. sIPSC frequency 60 min after LTP: 1.76 ± 1.2 Hz; Wilcoxon test, *X*^2^= 6.9, *p* = 0.01). Further, 1 h after iLTP induction, MNTB-evoked IPSCs could not be de-potentiated using an MNTB afferent stimulation pattern (LFS, 1 Hz, 15 min) previously shown to induce iLTD in naive slices (Kotak and Sanes, 2000). There was no significant difference between the IPSC amplitude at 60 min after LTP induction (541 ± 178 pA), as compared to the amplitude after an additional 15 min of 1 Hz/15 min stimulation (531 ± 163 pA; Wilcoxon test, *X*^2^ = 1.6, *p* > 0.1; *n* = 3).

**Figure 5:**
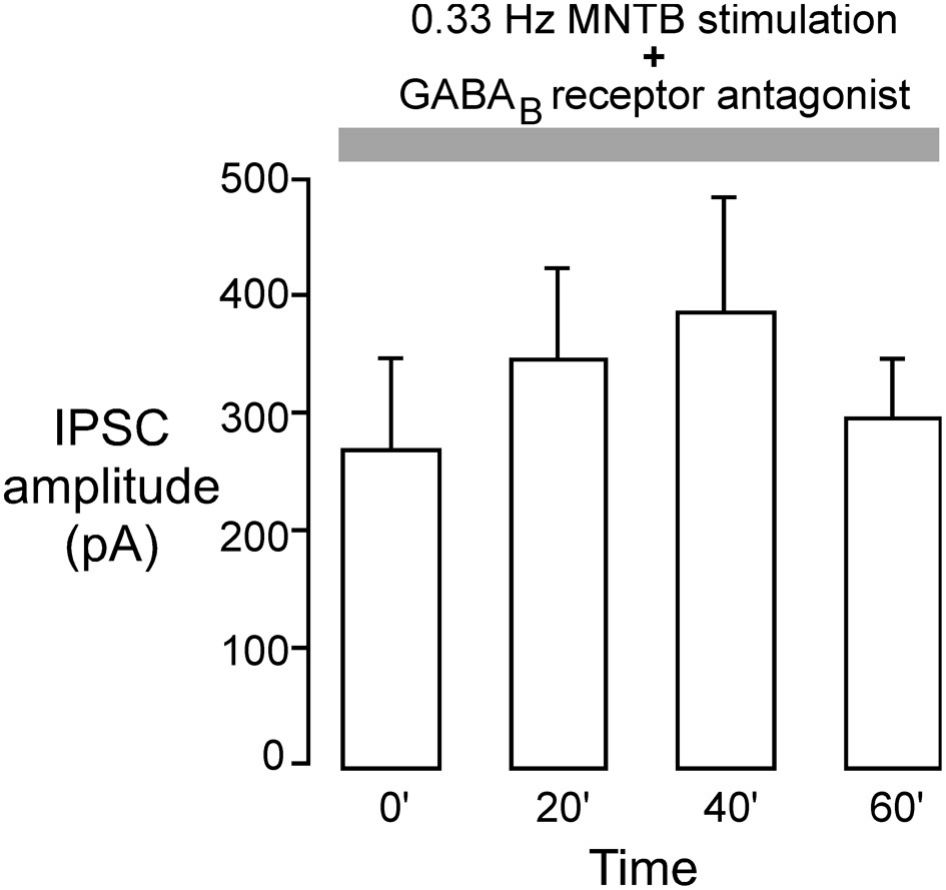
**GABA_B_ receptor blockade perturbs inhibitory iLTP**. MNTB was continuously stimulated at 0.033 Hz for 1 h while voltage clamp recordings were obtained from P12 to 15 LSO neurons in the presence of an ionotropic glutamate receptor antagonist (*V*_HOLD_ = 0 mV). The GABA_B_ receptor blocker, SCH-50911 (10 μM), was present throughout the recordings. Under these conditions, IPSC amplitudes did not show any significant change toward potentiation. Each bar represents the mean current (±s.e.m.) calculated at the beginning of the experiment, and at 20, 40, and 60 min.

Our previous study showed that iLTD in the LSO requires the activation of GABA_B_ receptors (Kotak et al., 2001). Therefore, we tested whether inhibitory LTP was also dependent on GABA_B_ receptor activation using a specific GABA_B_ receptor antagonist (SCH-50911) during induction of inhibitory LTP in P12–15 neurons. The MNTB was stimulated at the frequency that induced iLTP (0.033 Hz) in voltage clamp condition (Vh = 0 mV) while the slice was continuously exposed to 10 μM SCH-50911 (*n* = 4). As shown in Figure 5, the mean IPSC amplitude over an hour period did not display a significant change (initial IPSC: 260 ± 68 pA; IPSC at 60 min: 295 ± 56 pA, Wilcoxon test, *X*^2^ = 0.7, *p* = 0.8, *n* = 4). Thus, inhibitory LTP was blocked by the SCH compound.

## Discussion

The major finding of this study is that inhibitory MNTB synapses onto the LSO display an activity-dependent long-term potentiation (iLTP) following hearing onset (P12) but not prior to that. This contrasts with the induction of an equally robust iLTD before hearing onset (Kotak and Sanes, 2000). We do not imply that transition from no iLTD to iLTP occurs suddenly or precisely at hearing onset; rather, such plasticity mechanisms may develop gradually and may even become more pronounced during the several weeks thereafter as sound coding properties associated with ILD are consolidated in the LSO. We had proposed that iLTD before hearing onset may underlie the elimination of redundant inhibitory synapses in LSO and MSO (Sanes and Siverls, 1991; Sanes and Takàcs, 1993; Kotak and Sanes, 2000; Kapfer et al., 2002; Kim and Kandler, 2003). Even as the numbers of inhibitory boutons per axon are decreased (Sanes and Takàcs, 1993), the strength of individual existing connections becomes much stronger as revealed by the increase in the amplitude of inhibitory events (Sanes, 1993; Kim and Kandler, 2003). Therefore, we propose that the emergence of iLTP may be one form of plasticity to support inhibitory synapse stabilization and strengthening *in vivo*.

When coupled with MNTB stimulation, either postsynaptic depolarization alone, or activation of ipsilateral excitatory afferents alone, was not sufficient to induce iLTP under current clamp conditions. We propose two mechanisms that could mediate iLTP in an intact developing animal. First, sufficient postsynaptic depolarization could result from the synergistic activity of developing excitatory terminals and/or the co-release of glutamate from the MNTB terminals themselves (Gillespie and Kandler, 2005; Case and Gillespie, 2011; Alamilla and Gillespie, 2013). Second, it is possible that very low levels of glutamatergic afferent activity can activate postsynaptic metabotropic glutamate receptors, which trigger prolonged depolarizations and calcium entry by low levels of synaptic activity (Kotak and Sanes, 1995; Ene et al., 2003) that may be sufficient to support iLTP. This is consistent with our observation that IPSPs could be potentiated by the direct application of glutamate that may have led to calcium influx via activation of ionotropic as well as metabotropic glutamate receptors (Figure 2).

Our previous results have shown that GABA_B_ receptors are involved in the generation of iLTD (Kotak et al., 2001). Similarly, it appears that GABA_B_ receptors are involved in iLTP. Both sets of experiments were performed in voltage clamp conditions where the internal recording solution contained QX-314, which blocks the postsynaptic GABA_B_ receptor-gated K^+^ channel (Nathan et al., 1990; Andrade, 1991). The fact we did not observe iLTP in the presence of a selective antagonist (SCH-50911) could be consistent with either pre- or postsynaptic GABA_B_ receptor signaling (Figure 5). One reason for this is that blockade of the GABA_B_ receptor-gated potassium conductance by QX-314 in the pipette solution leaves open the possibility that other second messengers are involved (Kotak et al., 2001). In addition, the increase in sIPSC frequency that occurs during iLTP (Figure 4) suggests that a presynaptic mechanism may accompany postsynaptic strengthening.

The adjustments of auditory neuron response properties to dynamic range, frequency, or sound location during early life may well depend on activity-dependent synaptic plasticity mechanisms (Sanes and Constantine-Paton, 1983, 1985; Knudsen et al., 1984; Joseph and Hyson, 1993; Zhang et al., 2001; Magnusson et al., 2008). For mature LSO principal neurons to properly compute ILDs, the excitatory and inhibitory synapses must become precisely matched along the tonotopic axes during development (Moore and Caspary, 1983; Spangler et al., 1985; Cant and Casseday, 1986; Sanes and Rubel, 1988; Glendenning et al., 1991; Sanes and Siverls, 1991). Our observations raise the possibility that increased inhibitory synapse strength may permit these to stabilize during the time when specificity is achieved *in vivo*.

## Author contributions

Vibhakar C. Kotak and Dan H. Sanes conceived and designed the experiments, Vibhakar C. Kotak performed the experiments and analyzed the data, and Vibhakar C. Kotak and Dan H. Sanes wrote the paper.

### Conflict of interest statement

The authors declare that the research was conducted in the absence of any commercial or financial relationships that could be construed as a potential conflict of interest.
